# Human umbilical cord–derived mesenchymal stem cells improve chronic pancreatitis in rats via the AKT-mTOR-S6K1 signaling pathway

**DOI:** 10.1080/21655979.2021.1928441

**Published:** 2021-05-28

**Authors:** Lijun Kong, Xiangxiang Xu, Hewei Zhang, Yi Zhou, Hongjian Huang, Bicheng Chen, Zhenxu Zhou

**Affiliations:** a Key Laboratory of Diagnosis and Treatment of Severe Hepato-Pancreatic Diseases of Zhejiang Province, Zhejiang Provincial Top Key Discipline in Surgery, The First Affiliated Hospital of Wenzhou Medical University, Wenzhou, Zhejiang, China; b Ophthalmology Department, The Yiling Hospital of Yichang, Yichang, Hubei, China; c Department of Hernia and Abdominal Wall Surgery, The First Affiliated Hospital of Wenzhou Medical University, Wenzhou, Zhejiang, China

**Keywords:** Chronic pancreatitis, fibrosis, inflammation, mesenchymal stem cell, pancreatic stellate cells

## Abstract

Chronic pancreatitis (CP) is a progressive inflammatory disease. In clinical treatment, many patients cannot get a timely diagnosis and effective treatment due to the lack of early diagnosis indicators. Mesenchymal stem cells have immunomodulatory and anti-inflammatory effects, and have broad application prospects in treating auto-immune diseases and inflammatory diseases. This study aimed to clarify the mechanisms of human umbilical cord mesenchymal stem cells (HUCMSCs) in the treatment of CP. The rats were randomly divided into four groups, with six rats in each group: control group, CP group, CP + HUCMSCs–treated group I, and CP + HUCMSCs–treated group II. We evaluated the levels of inflammatory factors, fibrosis and apoptosis markers, detected the protein expression levels of AKT-mTOR-S6K1 and assessed histological changes of the pancreas. The results showed that HUCMSCs not only inhibited the secretion of inflammatory cytokines and activation of pancreatic stellate cells but also suppressed the apoptosis of acinar cells. Further investigation revealed that HUCMSCs noticeably suppressed the AKT-mTOR-S6K1 pathway in the pancreatic tissue of DBTC-induced CP. In addition, the therapeutic effect of HUCMSCs injected into the inferior vena cava and left gastric artery in the CP model was also observed, thus providing the basis for the clinical application of intervention measures.

## Graphical abstract

**Figure:**
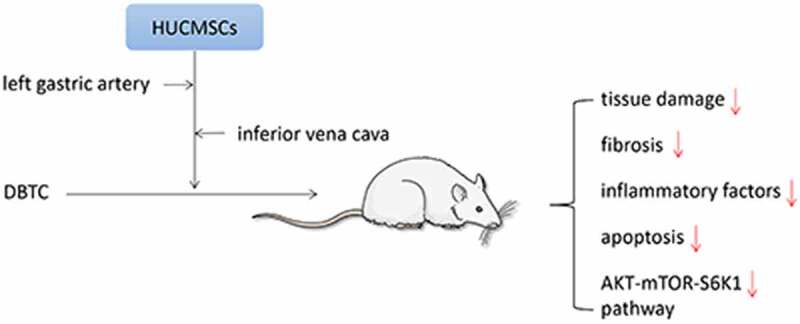

## Introduction

Chronic pancreatitis (CP) is a chronic disease of the pancreas accompanied by irreversible endocrine and exocrine dysfunction [1,2]. Although the incidence of CP is lower than that of acute pancreatitis, it has a far-reaching impact on the quality of life and is a recognized risk factor for pancreatic cancer [3,4]. CP is a common disease in gastroenterology, which can be divided into three major types: chronic auto-immune pancreatitis, chronic obstructive pancreatitis, and chronic calcifying pancreatitis. Although various methods have been used to treat CP, such as surgery, digestive enzyme supplements, and antioxidant therapy, the treatment of pancreatitis is still limited to relieving symptoms [5]. Many patients cannot get a timely diagnosis and effective treatment owing to the lack of indicators for early diagnosis. Therefore, the treatment of CP needs new ideas and methods.

The pathophysiological mechanisms of CP include the necrotic–fibrotic cycle caused by severe acute pancreatitis, followed by the activation and recruitment of inflammatory cells and the activation of pancreatic stellate cells (PSCs) and myofibroblast-like cells in the pancreatic exocrine region [6–9]. PSCs are continuously activated by pro-inflammatory cytokines, such as tumor necrosis factor-α (TNF-α), interleukin-1β (IL-1β) [10,11]. Activated PSCs proliferate and transform into myofibroblast-like cells, expressing α-smooth muscle actin (α-SMA) and synthesizing a large amount of extracellular matrix (ECM) protein, thus leading to the formation of fibrous tissue [12]. Therefore, inhibiting the growth of PSCs is an effective method for treating pancreatic fibrosis.

Mesenchymal stem cells (MSCs) are a promising candidate for cell therapy [13]. They secrete bioactive molecules with an anti-inflammatory effect, and have broad application prospects in treating auto-immune diseases and inflammatory diseases [14,15]. Unlike traditional molecular medicine, which focuses on targeting specific pathways, the stem cell therapy designed to disrupt many related mechanisms to gain therapeutic benefits [16]. A large number of preclinical studies have shown that cell therapy using MSCs can be used as a cell-based therapy for inflammatory diseases. Previous studies have shown that human umbilical cord mesenchymal stem cells (HUCMSCs) exert an anti-inflammatory effect by reducing the inflammatory response and cytokine production [17]. HUCMSCs have the advantages of wide source, convenient collection, and low immunogenicity. They can be regarded as immunodeficient cells due to the lack or low expression of major histocompatibility complex class II molecules and costimulatory molecules [18,19]. In early preclinical trials, HUCMSCs were used to rescue liver fibrosis and differentiate into hepatocytes in a rat model of chemical injury. In addition, HUCMSCs were injected into the rat model of xenotransplantation disease, and good implantation rate and functional results were obtained without immune rejection or tumor formation [20].

AKT-mTOR-S6K1 pathway is involved in mammalian cellular and physiological processes. Specifically, Akt is activated in response to extracellular growth factors and activates the downstream target mTOR. MTOR activated the expression of S6K1 cyclin. MTOR is an upstream kinase necessary for S6K1 activation and is important in cell development and function [21]. MTOR and S6K1 is involved in many physiological processes, including chemotaxis, cell migration and tumor cell invasion, translation, and cell growth [22,23]. In view of the importance of AKT-mTOR-S6K1 signaling in many diseases and aging cell functions, this study further elucidated that the molecular mechanism of HUCMSCs induced the inhibition of the AKT-mTOR-S6K1 signaling pathway.

This study aimed to observe the effects of HUCMSCs on the inflammatory process, fibrosis, and apoptosis in rats with CP. The therapeutic effect of dibutyltin dichloride (DBTC)-induced CP model on CP via inferior vena cava and left gastric artery injection was also explored. Our current results showed that both inferior vena cava and left gastric artery injection of HUCMSCs significantly improved chronic pancreatitis in rats. We further proved that AKT-mTOR-S6K1 was involved in the protective effect of HUCMSCs on chronic pancreatitis in rats.

## Material and methods

### Animals

The present study used 24 healthy adult male Wistar rats weighing 210 ± 10 g. Before surgery, the rats were fed for 1 week and acclimatized to the environment. The study was approved by the Institutional Animal Care and Use Committee of Wenzhou Medical University. The rats were randomly divided into four groups, with six rats in each group: control group (rats were injected with the same amount of physiological saline), CP group (CP was induced by tail vein injection of DBTC in rats), CP + HUCMSCs–treated group I (2,000,000 cells/kg HUCMSCs were injected into the inferior vena cava after DBTC-induced CP in rats after the third week), and CP + HUCMSCs–treated group II (2,000,000 cells/kg HUCMSCs were injected into the left gastric artery after DBTC-induced CP in rats after the third week). Except for the control group, others were given 10% ethanol per day for 10 weeks to induce pancreatic fibrosis. On the last day of the experiment, the rats were sacrificed under anesthesia to collect blood and pancreatic tissue samples.

## Cell culture

HUCMSCs were purchased from Cyagen Biosciences, Inc. and cultured in the Umbilical Cord MSC Growth Medium (Cyagen Biosciences, USA.). The cells were maintained at 37°C and 5% CO_2_ and passaged with 0.25% trypsin EDTA solution.

## Histopathological evaluation

The pancreatic tissue was fixed with 4% paraformaldehyde and cut into 5-µm paraffin sections. Sirius red staining was performed for 1 h, followed by Meyer hematoxylin staining at 25°C for 15 min. The pathological changes were observed under a light microscope, and the degree of pancreatic fibrosis was determined.

## Immunohistochemistry assays

The pancreatic tissue was fixed with 4% paraformaldehyde and cut into 4.5-µm paraffin sections. The paraffin sections were dewaxed in xylene for half an hour and then put in alcohol for gradient dehydration. The sample was boiled for 15 min with maximum power in the antigen repair buffer containing 0.01 mol/L citric acid–hydrochloric acid. The sample was blocked with hydrogen peroxide (3% H_2_O_2_) for 10 min to reduce the effect of endogenous peroxidase. Then, the fetal bovine serum was closed at 37°C for an hour. The cells were incubated overnight at 4°C with primary antibody against IL-6 (Abcam, USA), and IL-1β (Abcam, USA). The sections were further treated with secondary antibody and stained with diaminobenzidine (Beyotime, China). The nucleus was stained with hematoxylin, sealed with neutral resin, and observed under the light microscope.

## Western blot analysis

The pancreatic proteins were extracted by ultrasonic fragmentation of pancreatic tissue with radioimmunoprecipitation buffer (Beyotime, China), phosphatase inhibitor (Roche Diagnostics GmbH, USA), and phenylmethyl-sulfonyl fluoride (Beyotime Institute of Biotechnology, China) at a ratio of 100:10:1. The tissue lysate was then centrifuged at 12,000 rpm for 20 min at 4°C, and the supernatant was collected. Then, a BCA protein detection kit (Beyotime, China) was used for quantitative analysis. The same amount of protein (30 μg) was separated with 10% SDS‑PAGE and transferred to a polyvinylidene fluoride membrane. The membrane was sealed with 5% skimmed milk at 37°C for 1 h, and then incubated with primary and secondary antibodies. The first antibody of the following proteins was used: collagen type I, collagen type III, IL-6 (1:1000, Abcam, USA), IL-1β (1:1000, Abcam, USA), TNF-α (1:1000, Abcam, USA), BAX (1:1000, CST, USA), caspase-3 (1:1000, CST, USA), GAPDH (1:1000, CST, USA), mTOR (1:1000, CST, USA), phosphorylated (p)-mTOR (1:1000, CST, USA), S6K1 (1:1000, CST, USA), phosphorylated (p)-S6K1 (1:1000, CST, USA), P-53 (1:1000, CST, USA), phospho-p53 (Ser15) (1:1000, CST, USA), phospho-AKT (1:1000, CST, USA) and AKT (1:1000, CST, USA). Finally, the protein bands were displayed using VisionWorks imaging software (Eastman Kodak Company, Rochester, USA).

## ELISA quantification

Hyaluronic acid, fibronectin, and vitamin A were detected using an ELISA kit following the manufacturer’s instructions (Shanghai Boyun Biology, China).

## Statistical analysis

The aforementioned data were expressed as mean ± standard error of mean and analyzed using SPSS19.0 (IBM, Armonk, USA). The t test and one-way analysis of variance were used to analyze the statistics between groups, and the data were expressed as mean ± standard deviation. A P value <0.05 was considered statistically significant.

## Results

In this study, we aimed to explore the therapeutic effect of HUCMSCs on chronic pancreatitis and its potential mechanism. We measured the levels of inflammatory factors, fibrosis, apoptosis markers and evaluated the histological changes of the pancreas. At the same time, by comparing the therapeutic effects of injection of HUCMSCs into inferior vena cava and left gastric artery on chronic pancreatitis, the difference between the two treatments was discussed. Finally, we detected the changes of the expression level of AKT-mTOR-S6K1 in pancreatic tissue to provide a new idea for the effective treatment of chronic pancreatitis.

## CP rat model

**Figure 1.**
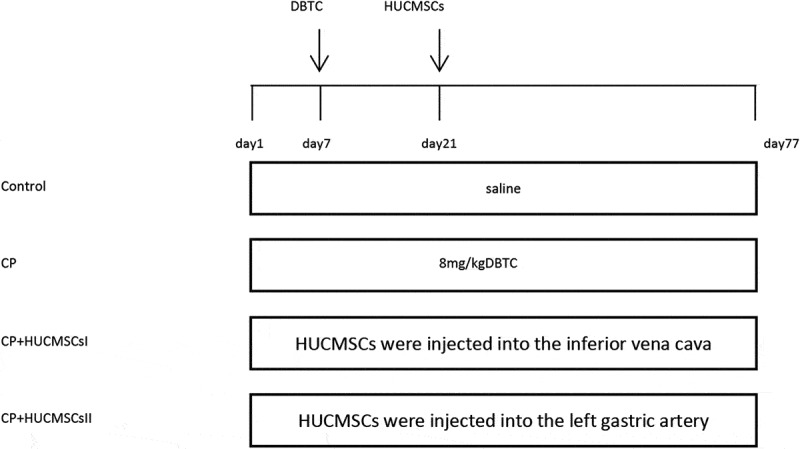
**Experiment schedule**. The rats were fed for 1 week to adapt to the environment. CP was induced by tail vein injection of DBTC. Except for the control group, others were given 10% ethanol per day for 10 weeks to induce pancreatic fibrosis. HUCMSCs were injected into the inferior vena cava and left gastric artery respectively in rats on the third week. DBTC, Dibutyltin chloride; HUCMSCs, human umbilical cord mesenchymal stem cells

**Figure 2.**
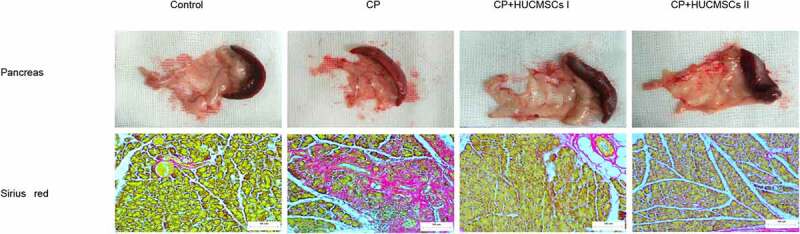
**In DBTC-induced pancreatic fibrosis, HUCMSCs reduced pancreatic tissue damage**. Morphology of pancreas. Sirius red staining sections were examined under an optical microscope (original magnification, 200×)

After acclimatizing the rats to the environment for 1 week, DBTC plus 10% ethanol was injected intravenously into the rats to induce CP (Figure 1). Compared with the control group, the pancreatic morphology in the CP group was abnormal, and the pancreatic tissue mass decreased. However, after pretreatment with HUCMSCs, the texture of the pancreas was soft, the adhesion to the surrounding tissue was poor, and the weight of the pancreas increased (Figure 2). Sirius red staining showed that the tissue structure around the islets in the control group was standard, and the pancreatic acinar cells were arranged closely. In the DBTC group, fibrosis, edema, and collagen deposition were observed. Pancreatic duct epithelial cells degenerated, and necrotic and inflammatory cells infiltrated widely in interlobular or acinar cells. HUCMSC treatment showed better pancreatic structure preservation and decreased Sirius Red–positive staining (Figure 2).

## HUCMSCs inhibited pancreatitis response

**Figure 3.**
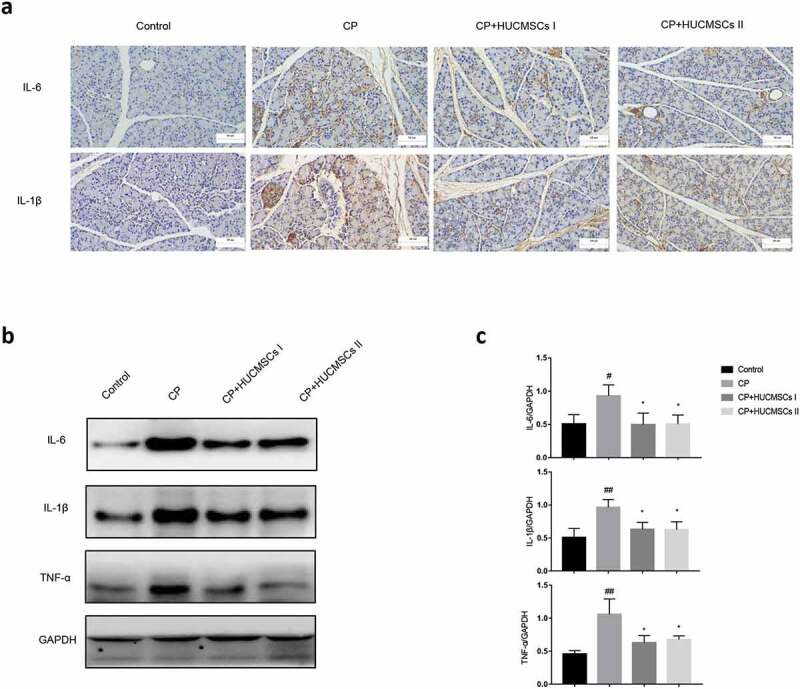
**HUCMSCs inhibited the expression of inflammatory factors in the pancreas**. Detection of the expression of IL-6 and IL-1β by (a) immunohistochemistry assays (original magnification, 200×) and (b-c) Western blot assay for the expression of IL-6, IL-1β and TNF-α. ^#^P < 0.05 versus the control group; *P < 0.05 versus the DBTC-induced CP model group

The expression of IL-1β and IL-6 in the pancreatic tissue significantly increased in the CP group. Compared with the CP group, the expression of inflammatory factors in the HUCMSC-treated group significantly reduced (Figure 3a). Western blot analysis showed that the expression of IL-6, IL-1β and TNF-α in the CP group significantly increased, while the expression of IL-6, IL-1β and TNF-α significantly decreased after HUCMSC treatment (Figure 3b).

## Treatment with HUCMSCs suppressed pancreatic fibrosis

**Figure 4.**
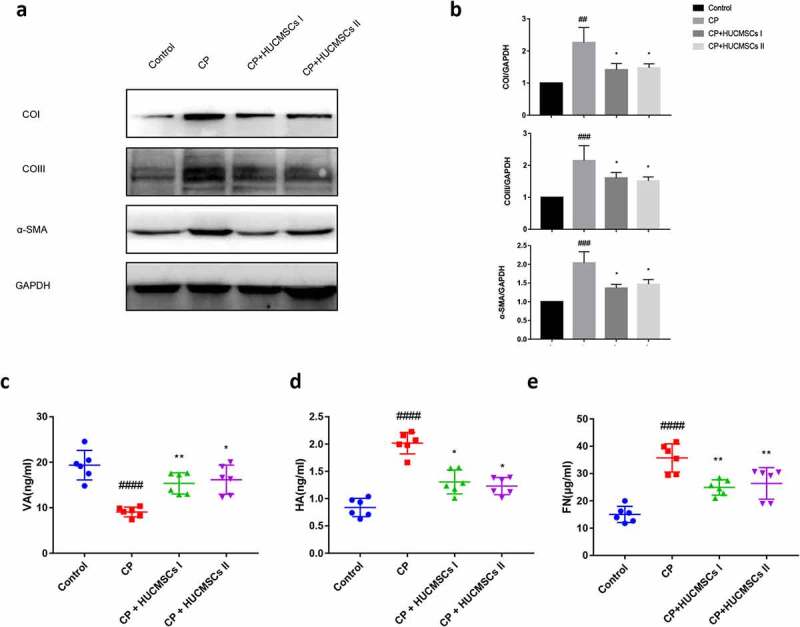
**HUCMSC treatment reduced fibrosis in CP**. (a–b) Expression of COI, COIII, and α-SMA was detected by Western blot analysis. (c–e) Contents of HA, VA, and FN in serum were measured by ELISA. #P < 0.05 versus the control group; *P < 0.05 versus the DBTC‑induced chronic pancreatitis model group; HA, Hyaluronic acid; VA, vitamin A; FN, Fibronectin; COI, collagen type I; COIII, collagen type III

The expression of α-SMA, collagen type I, and collagen type III in the pancreas was detected by immunohistochemical and Western blot analyses to verify the pancreatic fibrosis and collagen deposition in CP. In the control group, no collagen accumulation was found in the pancreatic cells, but it was noted in the vascular wall. Obvious collagen deposition and α-SMA expression were found in the CP group. However, the collagen deposition and the expression of α-SMA decreased significantly in the HUCMSC-treated group (Figure 4a, b). This finding indicated that HUCMSCs might be important in reversing pancreatic fibrosis in rats. In the quiescent phase, PSCs are lipid droplets rich in vitamin A. These perinuclear lipid droplets disappear when activated by inflammatory cytokines. Hyaluronic acid (HA) and Fibronectin (FN) are important components of the extracellular matrix. The serum levels of HA and FN were significantly lower in the HUCMSC-treated group than in the CP group, while vitamin A level was higher in the HUCMSC-treated group, suggesting that HUCMSCs affected the extracellular matrix metabolism (Figure 4c–e). No statistical difference was found between HUCMSC-treated group I and group II. This proved that HUCMSCs could alleviate pancreatic fibrosis and improve CP regardless of surgical procedure.

## HUCMSCs could decrease the apoptosis of pancreatic cells in DBTC-induced CP

**Figure 5.**
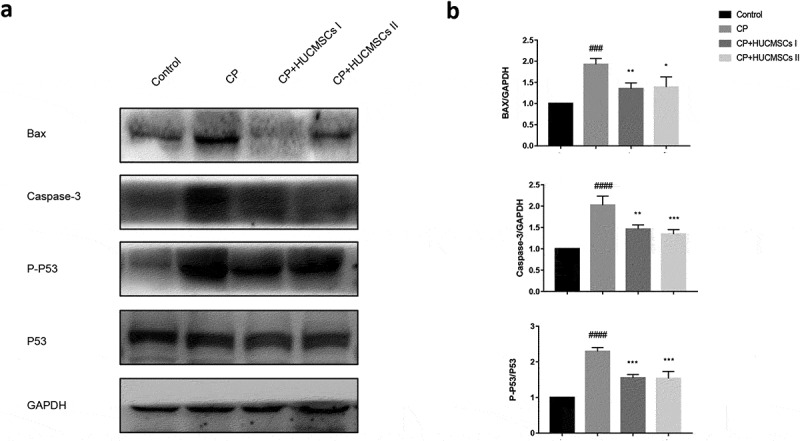
**HUCMSCs decrease the apoptosis of pancreatic cells in DBTC-induced CP**. (a–b) Western blot analysis was used to detect the expression of Bax, caspase-3, and p-53. #P < 0.05 versus the control group; *P < 0.05 versus the DBTC-induced chronic pancreatitis model group

Next, the effect of HUCMSC infusion on pancreatic apoptosis was measured. Compared with the control group, the apoptosis of pancreatic acinar cells in the CP group significantly increased after DBTC treatment. It was characterized by increased expression of apoptosis-related proteins Bax, p53, and caspase-3. However, the expression of apoptosis-related proteins decreased in the HUCMSC-treated group. No significant difference was found in the expression of apoptosis-related proteins between the two HUCMSC-treated groups (Figure 5a,b). This proved that HUCMSCs could improve DBTC-induced CP in rats whether it was the inferior vena cava or the left gastric artery.

## HUCMSCs suppressed the AKT-mTOR-S6K1 pathway in DBTC‑induced CP

**Figure 6.**
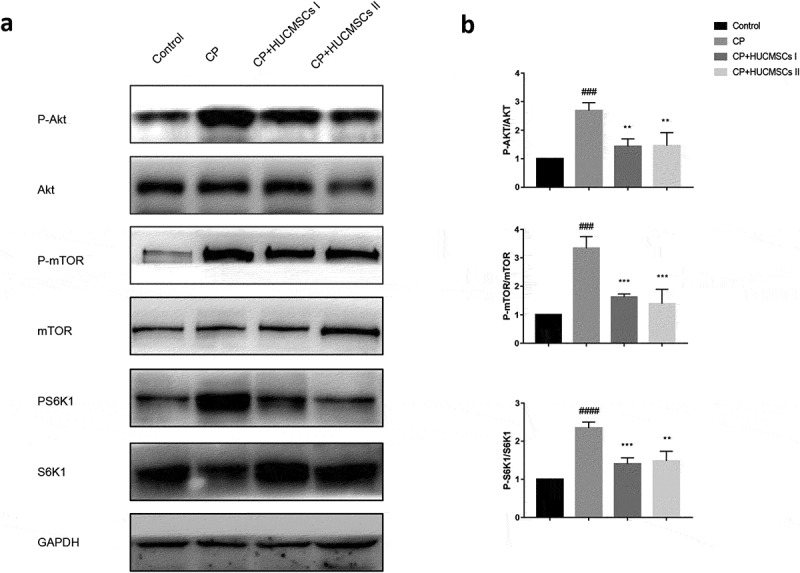
**HUCMSCs improve DBTC induced CP by inhibiting AKT-mTOR-S6K1 pathway**. Western blot analysis was used to detect the protein expression levels of (a-b) p-AKT, AKT, p-mTOR, mTOR, p-S6k1, and S6K1 . #P < 0.05 versus the control group; *P < 0.05 versus the DBTC-induced chronic pancreatitis modelgroup

The AKT-mTOR-S6K1 signal transduction activity was analyzed by Western blot analysis to explore the possible mechanism of stem cells in the treatment of chronic pancreatitis in rats. The results showed that the phosphorylation levels of AKT, mTOR, S6K1 and the ratio of p-AKT/AKT, p-mTOR/mTOR, p-S6K1/S6K1 in the CP group were significantly higher than those in the normal control group. However, the phosphorylation levels of AKT, mTOR, S6K1 and the ratio of p-mTOR/mTOR, p-s6k1/S6K1 decreased significantly after HUCMSC treatment (Figure 6a,Figure 6b). Based on these data, it was speculated that HUCMSC may improve chronic pancreatitis by inhibiting Akt-mTOR-S6K1 signaling pathway.

## Discussion

CP is a progressive inflammatory disease characterized by pancreatic gland atrophy with acinar cell damage, inflammatory cell infiltration, irregular fibrosis, and pancreatic secretion dysfunction [24]. The severity of CP depends on the local inflammation and the fibrosis degree of the pancreas [25]. In this study, HUCMSC infusion into the experimental rat model could greatly reduce pancreatic injury, significantly reduce the expression of inflammatory factors, and reduce fibrosis and cell death, thus inhibiting the progression of CP.

A large number of cytokines, such as IL-1β, IL-6, and TNF-α, are released during inflammation [26]. Among these pro-inflammatory molecules, TNF-α released by macrophages is one of the main factors for CP-induced inflammatory response, which can increase the release of other pro-inflammatory factors (such as IL-6) [14]. It increases the expression of chemokines and adhesion molecules, induces the recruitment of inflammatory cells, and eventually leads to pancreatic injury [27]. In this study, HUCMSC infusion could significantly reduce the expression of inflammatory factors in CP, reduce pancreatic injury, and protect the morphology of pancreas. This finding suggested that HUCMSCs might play a protective role in alleviating pancreatitis by neutralizing cytokines.

The morphology of PSCs in normal pancreatic tissue is round and static. The quiescent PSCs are characterized by the presence of vitamin A–rich lipid droplets [28]. However, these lipid droplets disappear from the cytoplasm during pancreatic injury or inflammation [29]. The serum vitamin A levels were measured and found to be the highest in the control group, followed by the HUCMSCs group. The level of vitamin A was significantly lower in the CP group than in the control and CP groups. Serum HA and FN are important markers of pancreatic fibrosis [30,31]. The ELISA results showed that the aforementioned indexes were significantly higher in the CP group than in the HUCMSC-treated groups, which, in turn, were higher compared with the control group. Inflammatory factors stimulate PSC to become fibroblast-like cells and secrete extracellular matrix proteins, including type 1 collagen and α-SMA, leading to pancreatic fibrosis [14]. ECM is necessary to maintain the normal tissue structure. In the pathological process, the balance of ECM synthesis and degradation changes, and the accumulation of ECM protein leads to tissue fibrosis [32]. Therefore, inhibiting PSC activation is one choice for treating pancreatic fibrosis in CP. In the DBTC-induced CP model, Western blot analysis showed that the expression of α-SMA, type I collagen, and type III collagen was upregulated, while their expression decreased in HUCMSCs [5]. This conclusion was verified by immunohistochemistry assays. HUCMSCs might inhibit pancreatic fibrosis by inhibiting the activation of PSCs. It might be a candidate drug for treating CP and pancreatic ductal adenocarcinoma associated with pancreatic fibrosis.

Bax is a member of the proapoptotic Bcl-2 family. It can interact with voltage-dependent ion channels in mitochondria and mediate the release of cytochrome c, which induces apoptosis [5]. P53 upregulate the expression of Bax and finally activate caspase-3 during apoptosis. The Western blot analysis showed that the expression levels of Bax, p-p53, and caspase-3 increased in the CP group, but decreased after HUCMSC treatment.

The AKT-mTOR-S6K1 pathway is related to the proliferation, apoptosis, metabolism and autophagy [33]. Akt plays an important role in cell survival and apoptosis. mTOR is a serine/threonine protein kinase that regulates protein synthesis. The activation of mTOR pathway involves many aspects of molecular and cellular biology. It is important in TNF-α-induced inflammatory cascade and promotes the occurrence of chronic inflammation–induced cancer. S6K1 is also a serine/threonine kinase. The phosphorylation of mTOR activates its function to promote the mRNA translation of target genes. Previous studies reported that the mTOR/S6K1 pathway promoted inflammation and tumorigenesis through the upregulation of vascular endothelial growth factor and subsequent angiogenesis [34]. In this study, HUCMSCs significantly reduced AKT, mTOR and S6K1 phosphorylation, suggesting that HUCMSCs could successfully inhibit the AKT-mTOR-S6K1 pathway and reduce DBTC-induced CP in rats. The results of this study provided new insights into the role of AKT, mTOR and S6K1 in the regulation of CP, and helped understand how HUCMSCs regulated the mTOR signal transduction system.

The therapeutic efficacy of pharmacological treatment usually depends on whether the appropriate drug concentration is reached [35]. The artery and vein close to the lesion site were selected at the injection site to maximize the drug concentration in the pancreas and around the pancreas. So as to accelerate the entry of the drug into the lesion site and improve the maintenance time of the drug concentration. Recent studies reported that the effect of acute pancreatitis through regional arterial infusion was better than that of intravenous therapy in clinical practice [36]. Interestingly, previous studies demonstrated that adipose-derived MSCs could improve CP in rats through the PI3K-Akt-mTOR pathway. The injection of adipose-derived MSCs into the inferior vena cava and left gastric artery proved that the two groups had no significant difference between them [37]. Therefore, this study explored the therapeutic effect of HUCMSCs injected into the inferior vena cava and left gastric artery in patients with CP. The effects of HUCMSCs on pancreatic inflammation, fibrosis, and apoptosis were analyzed in these patients. In terms of providing preclinical validation for the treatment using HUCMSCs and their derived tissues in disease models, the results showed no significant difference between the two groups.

## Conclusion

The assessment and treatment of CP are challenging, and most patients are still symptomatic after treatment. However, as the understanding of CP continues to evolve, some new insights have improved treatment to prevent the onset of CP, slow its progression, and possibly reverse its fibro inflammation. A large number of cell and animal model studies have proved the safety and effectiveness of HUCMSCs in vivo. The results of this study further provided the basis for the fate of injected stem cells and their potential impact on the pancreatic microenvironment, and enriched the understanding of tissue repair mechanisms. Further detailed experiments are needed to verify how to translate it into clinical application. It is still a long way to go before HUCMSCs can be used in clinical treatment.

## Highlights

1. HUCMSCs effectively reduced the DBTC-induced pancreatic damage.
2. HUCMSCs noticeably suppressed the AKT-mTOR-S6K1 pathway in chronic pancreatitis.
3. The therapeutic effect of HUCMSCs injected into inferior vena cava and left gastric artery on chronic pancreatitis was not statistically significant.
